# Energy-saving and pricing decisions in a sustainable supply chain considering behavioral concerns

**DOI:** 10.1371/journal.pone.0236354

**Published:** 2020-08-04

**Authors:** Bin Chen, Wenying Xie, Fuyou Huang, Xinyang Li

**Affiliations:** 1 Institute of Transportation Development Strategy & Planning of Sichuan Province, Chengdu, Sichuan, China; 2 School of Transportation and Logistics, Southwest Jiaotong University, Chengdu, Sichuan, China; Sunway University, MALAYSIA

## Abstract

With the rising environmental concerns among consumers all over the world, sustainability has received considerable attention, and numerous enterprises are adopting various practices such as investing in energy-saving to improve sustainability in supply chains. However, many previous researches always assume that decision makers are perfectly rational and neglect the behavioral concerns of decision makers. This paper considers a two-stage sustainable supply chain with behavioral concerns in order to develop more realistic models, and mainly focuses on the energy-saving and pricing decisions in the decentralized system, as well as how to improve energy-saving level and profits. We develop decentralized decision-making models under two types of behavioral concerns: fairness concern and risk aversion, and derive the optimal strategy for each member with a Stackelberg game in which the manufacturer acts as the leader. The effect of the behavioral concerns on the optimal decisions and corresponding profits is discussed in detail. Theoretical analysis verified by numerical experiments shows that the fairness behavior always causes a negative effect on the manufacturer, total supply chain, and energy conservation, while it could benefit the retailer in profits. The risk aversion behavior always benefits the manufacturer, total supply chain, and energy conservation, whereas it could make the retailer suffer. Note that both the optimal energy-saving level and corresponding profit of the total supply chain under two types of behavioral concerns are lower than that in the centralized system, thereby we propose a revenue-cost-sharing contract to coordinate the supply chain, under which both the manufacturer and the retailer can achieve a win-win outcome and the energy-saving level can be improved. In addition, some managerial implications through our analytical and numerical results are summarized in this paper.

## Introduction

During the past few decades, a series of environmental disasters have been caused by drastic changes in climate and air pollution [1], and human survival environment is being severely damaged [2]. For instance, the combined heating influence of all major greenhouse gases increases by 43% between 1990 and 2019. Nowadays, global concerns about environmental protection and sustainability have increased substantially [3, 4]. Governmental organizations, media, activists and consumers are stimulating enterprises to extend their responsibility and consider sustainability in their decisions and operations, which is a significant challenge for businesses [5]. Meanwhile, world energy consumption has experienced a significant increase during the previous decades, from shifting from 3700 Mtoe in 1965 to 13,147 Mtoe in 2015, and thereby energy shortages has become increasingly prominent [6]. In order to address energy shortages and pollution reduction issues, energy saving has become an important mean to promote sustainable development [7, 8]. Many countries and supranational organizations announce environmental protection regulations to reduce greenhouse gas emissions, and induce companies to adopt practical energy-saving strategies to enhance energy efficiency [9]. For example, some countries in the European Union proposed an energy-saving plan to reduce energy consumption by 17% over the next three decades [10]. The United States Government offered a federal subsidy program for electric vehicles, and consumers can receive a one-time bonus of up to a maximum of $7500 (USD) upon purchasing [11]. The Chinese government encouraged consumers to purchase energy-saving products such as variable-frequency air conditioners and energy-saving automobiles by providing subsidies [12].

On the other hand, many consumers recognize that their consumption affects the environment, and the environmental awareness is changing consumer’s purchasing behavior [13]. An increasing number of consumers are willing to buy energy-saving products or environmentally friendly products than before [14–16]. For instance, according to a survey conducted by Accenture, more than 80% of interviewees stated that they would pay attention to the sustainability of products when making their purchasing decisions [17]. As carried out by European Commission in 2008, there are 75% of Europeans are ready to buy green products even if they cost a little more, up from 31% in 2005 [15]. In sum, products with high energy-saving level would have a higher market share, reputation and market value [18]. Therefore, increasing public environmental awareness and pressure from governments are attracting enterprises toward environmental protection, many socially responsible manufacturers have been actively investing in energy saving and producing environmentally friendly products. However, investment in energy-saving is costly, thus the problem about how to decide energy-saving level and price is paid increasing attention in academia and industry.

In addition, a series of empirical and experimental studies in behavioral economics have shown that supply chain managers may involve bounded rationality and have different behavioral concerns when they make decisions [19–22], and the behavioral concerns can significantly influence optimization in supply chains. However, many existing researches about energy-saving and pricing decisions assume decision makers are perfectly rational. Motivated by this observations and discussions, we study a sustainable supply chain composed of a socially responsible manufacturer who invests in technologies to improve energy-saving level and a retailer who involves behavioral concerns such as fairness concern and risk aversion. We explore the energy-saving and pricing decisions under three scenarios: centralized system, decentralized system without coordinating contract and decentralized system with revenue-cost-sharing contract.

The main contributions of this work are as follows. First, we establish a performance benchmark by the centralized system, and obtain the system-wide optimal energy-saving level, retail price and corresponding profit. Second, we develop decentralized decision-making models under two types of behavioral concerns, and derive the optimal strategy for each member with a Stackelberg game in which the manufacturer acts as the leader and the retailer is the follower. Then, we discuss the effect of the two types of behavioral concerns on the optimal equilibriums and corresponding profits in detail, and compare the optimal energy-saving level, retail price and corresponding profits under three cases: perfect rationality, behavioral concerns and centralized system. We find that the efficiency of energy-saving investment plays a very critical role in energy-saving and pricing decisions, and the change of the optimal equilibriums with respect to the coefficients of behavioral concerns depends on the efficiency of energy-saving investment. Also, it is noted that whatever behavioral concerns the retailer involves, both the optimal energy-saving level and corresponding profit in the decentralized system are lower than that in the centralized system. Third, we propose a revenue-cost-sharing contract to coordinate the supply chain so as to enhance the overall environmental and commercial performances of the supply chain. In addition, we provide some managerial implications for sustainable supply chains considering energy-saving investment and behavioral concerns.

The rest of this paper is organized as follows. The relevant literature is reviewed in next section. Section 3 provides the problem description, assumptions and notations, and gives the centralized decisions of the supply chain to establish a performance benchmark. In Section 4, we develop decentralized decision-making models under two types of behavioral concerns, and discuss the effect of the two types of behavioral concerns on the optimal equilibriums and corresponding profits in detail. A revenue-cost-sharing contract is proposed to develop supply chain coordination models in Section 5. In Section 6, we use several numerical experiments to verify the theoretical analysis obtained in the previous sections. Section 7 concludes this paper and provides some managerial insights. The detailed proofs of propositions and corollaries in this paper appear in S1 Appendix.

## Literature review

The three streams of research of energy-saving and pricing decisions, fairness concern and risk aversion are closely related to this study. In this section, we provide a brief review of the relevant literature and highlight the differences between this study and the existing literature.

Currently, there is a growing literature focusing on the energy-saving and pricing decisions in sustainable supply chains. Javadi et al. [10] studied the optimal pricing decisions in a dual-channel supply chain consisting of a manufacturer and a retailer under different flexible return and energy-saving regulations. Xie [23] investigated the optimal strategies in a two-echelon supply chain where a manufacturer decides the energy-saving level of product and a retailer determines the retail price of product, and explored the coordination issue of the supply chain by wholesale price and profit sharing schemes and a lump sum transfer contract. Then, Xie [24] investigated the optimal energy-saving decisions and cooperative strategies in a decentralized supply chain with two competing suppliers and a manufacturer. Xie et al. [25] analyzed the optimal decisions on the energy efficiency of main engines and the price of the shipbuilding service in a sustainable shipbuilding supply chain, and suggested that technological innovations should be advocated to reduce the fixed costs for effective improvements of the energy efficiency of main engines. Hafezalkotob [26] developed price-energy-saving competition and cooperation models for two green supply chains under government financial intervention, and examined the energy-saving and pricing decisions under four supply-chain configurations. Zhou and Huang [27] studied two types of contracts for energy-saving products in a monopoly with the government’s budget constraint, and discussed the energy-saving level and price decisions of enterprises. Yi and Li [28] established a Stackelberg game model to study the cooperation of a supply chain in energy saving and emissions reduction, where the retailer decide a cost-sharing contract and the retail price, and the manufacturer decides the energy-saving level, emissions-reduction level and wholesale price. However, it should be noted that all the aforementioned researches assume decision makers are perfectly rational.

Fairness concern is one of typical behavioral concerns. When a company involves fairness behavior, it will not only care about its monetary payoff but also the fairness outcome of profit allocation in the supply chain [29]. For example, if a manufacturer is powerful relative to a retailer, the manufacturer who manages the supply chain is the leader to provide contacts, and the retailer is the follower. The fair-minded retailer would decrease its utility, since the profit derived from the total supply chain could be less than that of the manufacturers [19]. In fact, the seminal work on supply chain management with fairness concern is introduced in a conventional two-echelon supply chain by Cui et al. [19], and this study is subsequently extended in different scenarios such as closed-loop supply chains [30], dual-channel supply chains [31] and sustainable/green supply chains [32, 33]. In sustainable/green supply chains, Zhou et al. [34] examined how the co-op advertising contract and the co-op advertising/emission reduction cost sharing contracts impact the low-carbon supply chain’s optimal decisions and coordination, and discussed how the optimal decisions and coordination change when a retailer has fairness concern. Li et al. [29] incorporated a retailer’s fairness concern into carbon emission reduction game models in a low-carbon supply chain, and analyzed the effects of fairness concern on pricing and carbon emission reduction, as well as the performance of a revenue-sharing contract in the supply chain. Du et al. [35] examined the impact of fairness concern on sustainable green technology innovation development in a two-stage supply chain. Zhang et al. [36] explored the joint effect of consumer environmental awareness and fairness concern on environmental quality decision and pricing in a green supply chain.

Risk aversion is another common and important behavioral concern. Many existing researches indicate that a firm is usually risk-averse when it faces uncertainties such as random demand [20, 37]. The mean-variance (MV) theory, established by Markowitz in the 1950s, has been widely applied to conduct risk analysis in operations management [38], and which optimizes the problem with consideration of both payoff and risk where payoff is captured by the expected profit and risk is measured by the variance of profit [39]. There are a number of studies which adopt the MV theory to analyze how the risk aversion affects the decisions of enterprises in traditional supply chain management [40, 41], whereas the works on sustainable/green supply chains with risk-averse firms are limited. Choi and Chiu [39] developed mean-downside-risk and MV newsvendor models for sustainable fashion retailing under both the exogenous and endogenous retail price decision cases. Xie et al. [42] investigated the optimal selection of cleaner products in a green supply chain under two different supply chain structures: a vertically integrated and a decentralized setting, in which the MV approach is used to model the risk aversion of each member. Bai and Meng [43] considered a two-echelon supply chain composed of two competing manufacturers and one retailer under investment in carbon emission reduction, and discussed the impact of risk aversion on the optimal strategies of supply chain members. Lai et al. [44] explored sustainability investment in a maritime supply chain with risk behavior and information sharing, they proposed a two-period game-theoretic model in a MV framework, and investigated the effect of risk aversion and information sharing on sustainability investment.

To the best of our knowledge, there are no studies that address the energy-saving and pricing decisions in a same sustainable supply chain with different behavioral concerns such as fairness concern and risk aversion. This paper aims to address the gap in the literature. Compared with the existing literature, we detailedly discuss the impact of the fairness concern and risk aversion on the optimal strategies in a same sustainable supply chain, and investigate the supply chain coordination issue with a revenue-cost-sharing contract. Moreover, we gain more interesting finds and managerial insights.

## Problem description and performance benchmark

We consider a two-stage supply chain consisting of a socially responsible manufacturer and a retailer involving behavioral concerns in a single period, where the manufacturer produces energy-saving products (e.g., energy-efficient air conditioners or refrigerators) with marginal production cost *c* and sells them to the retailer at wholesale price *w*, then the retailer resells energy-saving products to end consumers at retail price *p* in the selling season. Due to the social responsibilities and the rising environmental consciousness of customers, the manufacturer has to invest in advanced facilities/technologies to improve energy-saving level during the production process. Consequently, the market demand would generally increase as the product’s energy-saving level *e* goes up. According to the literature [24, 44], the market demand is stochastic in nature and dependent on energy-saving level of product and retail price, which can be expressed as

$$D=\tilde{a}-\alpha p+\beta e$$ (1)

where $\tilde{a}$, *α*(*α*>0) and *β*(*β*>0) are the potential intrinsic demand, consumer’s sensitivity parameters in retail price and energy-saving level, respectively. In order to capture uncertainty in market demand resulting from changes in economic and business conditions, $\tilde{a}$ is assumed to be a random variable and shown as $\tilde{a}=a+\zeta$, where *a* is the mean of the potential intrinsic demand, and *ζ* follows the normal distribution with *E*(*ξ*) = 0 and *Var*(*ζ*) = *σ*^2^. To avoid trivialities, we have *p*>*w*>*c* and *a*>*αp*>*αc*. Thus, the expected demand function is written as

d=a−αp+βe (2)

We let *C*(*e*) be the cost of energy-saving investment, which is an increasing convex function of the energy-saving level *e*, namely, *C*(*e*)>0, *C*'(*e*)>0 and *C*"(*e*)>0. Generally speaking, as the energy-saving level increases, it becomes increasingly more expensive. Since the quadratic function not only captures the phenomenon of increasing marginal cost but also reflects the characteristic that the investment cost of the initial energy level per unit is decreasing, we let *C*(*e*) = *ke*^2^/2. *k* is the coefficient of energy-saving investment, which represents the impact of energy-saving on cost. It is common to use the form of quadratic function to describe the quantitative relationship between the environmental improvement and the corresponding expenditure in existing literature [15, 28, 32].

The manufacturer plays a leading role in the supply chain, and makes decisions based on the principle of maximizing expected profit as rational economic people. The retailer acts as a follower, and aims to maximize expected utility since the retailer involves behavioural concerns. In this study, the energy-saving level, wholesale price and retail price are decision variables and other variables are exogenous variables, known to both the manufacturer and the retailer. We use subscript *m*, *r* and *sc* to successively denote the manufacturer, retailer and total supply chain, respectively. The second subscript letter *n* denotes the perfect rationality case, *λ* denotes the fairness concern case, and *η* denotes the risk aversion case. Also, we use superscript *I*, *D* and *R* to successively denote the centralized system, the decentralized decision-making model, and the revenue-cost—sharing contract coordination model, respectively.

Notations used in this paper are listed in Table 1.

**Table 1:** Notations.

Notation	Implication
*c*	production cost per unit of product
*w*	wholesale price per unit of product
*p*	retail price per unit of product
*e*	energy-saving level
$\tilde{a}$	potential intrinsic demand
*a*	mean of the potential intrinsic demand
*α*	consumer’s sensitivity parameter in retail price
*β*	consumer’s sensitivity parameter in energy-saving level
*σ*	standard deviation of the potential intrinsic demand
*k*	coefficient of energy-saving investment
*λ*	fairness concern parameter of the retailer
*η*	risk aversion degree of the retailer
*φ*	revenue-sharing coefficient
*θ*	cost-sharing coefficient
*π*	profit function
*U*	utility function

In what follows, we use the centralized system to establish a performance benchmark. In the centralized case, the manufacturer and the retailer would jointly operate as one decision-maker to decide the energy-saving level *e*^*I*^ and the retail price *p*^*I*^ to maximize the system-wide expected profit, which aligns with the extant literature [21, 32]. Let $E\pi_{sc}^{I}$ denote the expected profit of the centralized supply chain, we have

$$E\pi_{sc}^{I}=(p^{I}-c)(a-\alpha p^{I}+\beta e^{I})-\frac{1}{2}k{\left(e^{I}\right)}^{2}$$ (3)

Differentiating with respect to *e*^*I*^ and *p*^*I*^ gives

$$\frac{\partial E\pi_{sc}^{I}}{\partial e^{I}}=\beta (p^{I}-c)-ke^{I}$$ (4)

$$\frac{\partial E\pi_{sc}^{I}}{\partial p^{I}}=a-2\alpha p^{I}+\beta e^{I}+\alpha c$$ (5)

The Hessian matrix of the expected profit of the centralized supply chain with respect to the energy-saving level and the retail price is

$$H^{I}(e^{I},p^{I})=\left|\begin{array}{l} -2\alpha \;\beta \\ \beta \;-k \end{array}\right|=2\alpha k-\beta^{2}$$ (6)

When 2*αk*−*β*^2^>0, the Hessian matrix is negative definite. This implies that $E\pi_{sc}^{I}$ is jointly concave in *e*^*I*^ and *p*^*I*^, there exist unique optimal solutions (*e*^I*^,*p*^I*^) which jointly determined by $\partial E\pi_{sc}^{I}/\partial e^{I}=0$ and $\partial E\pi_{sc}^{I}/\partial p^{I}=0$. Thus, we get

$$p^{I*}=\frac{ak+\alpha kc-\beta^{2}c}{2\alpha k-\beta^{2}}$$ (7)

$$e^{I*}=\frac{(a-\alpha c)\beta }{2\alpha k-\beta^{2}}$$ (8)

Note that, since *p*^I*^>*c*, we get *p*^I*^−*c* = *k*(*a*−*αc*)/(2*αk*−*β*^2^)>0. Thus, 2*αk*−*β*^2^>0 always holds because *a*−*αc*>0. That is, there always exist unique optimal solutions in the centralized system. Furthermore, we can naturally obtain *β*^2^/*k*<2*α*, and *β*^2^/*k* can be interpreted as the efficiency of energy-saving investment. *β*^2^/*k*<2*α* indicates that the efficiency of energy-saving investment is limitary and the cost of improving energy-saving level is high in practical applications. This is consistent with the existing literature [32, 45].

Therefore, the maximum expected profit of the supply chain can be achieved at *p*^I*^ and *e*^I*^. In this case, the optimal expected profit of the supply chain is

$$E\pi_{sc}^{I*}=\frac{k{\left(a-\alpha c\right)}^{2}}{2(2\alpha k-\beta^{2})}$$ (9)

Next, we will explore the optimal decisions of both the manufacturer and the retailer with different behavioral concerns in the decentralized system, and discuss what strategy to choose by comparing each of them with the benchmarking case.

## Decentralized decision-making model

In the decentralized system, the manufacturer and the retailer are independent self-interested firms. There is a Stackelberg game in which the manufacturer acts as the leader and the retailer acts as the follower. We will analyze the optimal strategy for each partner via backward induction to find the equilibriums of the Stackelberg game. We first explore the retailer’s problem of determining retail price given the energy-saving level and wholesale price, and then we investigate the manufacturer’s strategy on the energy-saving level and wholesale price.

In the decentralized system, the profit functions of the manufacturer and the retailer are given by

$$\pi_{m}^{D}=(w^{D}-c)(\tilde{a}-\alpha p^{D}+\beta e^{D})-\frac{1}{2}k{\left(e^{D}\right)}^{2}$$ (10)

$$\pi_{r}^{D}=(p^{D}-w^{D})(\tilde{a}-\alpha p^{D}+\beta e^{D})$$ (11)

Thus, the profit function of the total supply chain in the decentralized system is

$$\pi_{sc}^{D}=\pi_{m}^{D}+\pi_{r}^{D}=(p^{D}-c)(\tilde{a}-\alpha p^{D}+\beta e^{D})-\frac{1}{2}k{\left(e^{D}\right)}^{2}$$ (12)

### Fairness concern scenariro

In this subsection, we investigate the scenario of the supply chain with a fair-minded retailer. The current research introduces profit differences into the utility function of decision makers to characterize fairness concern, and the equitable reference point of the retailer is the manufacturer’s profit. We introduce the coefficient of fairness concern to describe the effect of the fairness concern brought about by the profit difference of the manufacturer and the retailer. When the retailer’s profit is greater than the profit of the manufacturer, its utility will be increased, otherwise, its utility will be reduced. In particular, the formulation of fairness would narrow down the profit gap between the manufacturer and retailer [46]. Following the literature [19, 30, 32], the utility function of the retailer with fairness concern can be expressed as follows $U_{r\lambda }^{D}=\pi_{r}^{D}-\tilde{\lambda }(\pi_{m}^{D}-\pi_{r}^{D})$, where $\tilde{\lambda }\in (0,\infty )$. For simplicity, we let $\lambda =\tilde{\lambda }/\left(1+\tilde{\lambda }\right)$, the utility function of the retailer with fairness concern can be rewritten as

$$U_{r\lambda }^{D}=\pi_{r}^{D}-\lambda \pi_{m}^{D}$$ (13)

where *λ* represents the retailer’s fairness concern coefficient, which satisfies 0<*λ*<1. Especially, *λ* = 0 corresponds to the rational economic people scenario.

Thus, when the retailer involves fairness concern, the expected utility of the retailer is

$$EU_{r\lambda }^{D}=[p_{\lambda }^{D}-(1+\lambda )w_{\lambda }^{D}+\lambda c](a-\alpha p_{\lambda }^{D}+\beta e_{\lambda }^{D})+\frac{1}{2}\lambda k{\left(e_{\lambda }^{D}\right)}^{2}$$ (14)

Then, we will obtain the optimal equilibriums of supply chain members via backward induction. The detailed proofs of propositions and corollaries in this paper appear in Appendix A.

**Proposition 1.** *In the decentralized supply chain with a fair-minded retailer*, *the optimal energy-saving level and wholesale price are*

$$e_{\lambda }^{D*}=\frac{(a-\alpha c)\beta }{4\alpha k(1+\lambda )-\beta^{2}}$$ (15)

$$w_{\lambda }^{D*}=\frac{2ak+2\alpha kc(1+2\lambda )-c\beta^{2}}{4\alpha k(1+\lambda )-\beta^{2}}$$ (16)

and the optimal retail price is

$$p_{\lambda }^{D*}=\frac{3\alpha k(1+\lambda )+c[\alpha k(1+\lambda )-\beta^{2}]}{4\alpha k(1+\lambda )-\beta^{2}}$$ (17)

Proposition 1 shows that the fairness behavior of the retailer affects not only the retail pricing but also the decisions of the manufacturer on the energy-saving level and wholesale price. Then, we have the following corollary.

**Corollary 1.** *In the decentralized supply chain with a fair-minded retailer, we have* $\partial e_{\lambda }^{D*}/\partial \lambda <0$, $\partial w_{\lambda }^{D*}/\partial \lambda <0$ and $\partial p_{\lambda }^{D*}/\partial \lambda <0$.

Corollary 1 shows that the intensified fairness of the retailer would decrease the energy-saving level, wholesale price and retail price. That is, all the optimal energy-saving level, wholesale price and retail price in a supply chain with a fair-minded retailer are lower than that of the fair-neutral scenario. This implies that when the retailer involves fairness behavior, the retailer will reduce the energy-saving investment. Accordingly, the energy consumption per unit product would increases, and the supply chain would suffer in terms of sustainability. Let *λ* = 0, we easily get the optimal decisions of the fair-neutral scenario, the optimal energy-saving level, wholesale price and retail price are characterized as $e_{n}^{D}=\beta (a-\alpha c)/\left(4\alpha k-\beta^{2}\right)$, $w_{n}^{D}=\left(2ak+2\alpha kc-\beta^{2}c\right)/\left(4\alpha k-\beta^{2}\right)$ and $p_{n}^{D}=\left(3\alpha k+\alpha kc-\beta^{2}c\right)/\left(4\alpha k-\beta^{2}\right)$, respectively. Furthermore, in the supply chain with a fair-minded retailer, the optimal expected profits of the manufacturer, retailer, and total supply chain can be given by

$$E\pi_{m\lambda }^{D*}=\frac{k{\left(a-\alpha c\right)}^{2}}{8\alpha k(1+\lambda )-2\beta^{2}}$$ (18)

$$E\pi_{r\lambda }^{D*}=\frac{k^{2}\alpha {\left(a-\alpha c\right)}^{2}(1+\lambda )(1+3\lambda )}{{\left[\beta^{2}-4\alpha k(1+\lambda )\right]}^{2}}$$ (19)

$$E\pi_{sc\lambda }^{D*}=E\pi_{m\lambda }^{D*}+E\pi_{r\lambda }^{D*}=\frac{k{\left(a-\alpha c\right)}^{2}[6\alpha k{\left(1+\lambda \right)}^{2}-\beta^{2}]}{2{\left[\beta^{2}-4\alpha k(1+\lambda )\right]}^{2}}$$ (20)

The optimal expected utility of the retailer is

$$EU_{r\lambda }^{D*}=\frac{k{\left(a-\alpha c\right)}^{2}[\beta^{2}\lambda +2\alpha k{\left(1+\lambda \right)}^{2}]}{2{\left[\beta^{2}-4\alpha k(1+\lambda )\right]}^{2}}$$ (21)

Conducting the sensitivity analysis, we further derive the following corollary.

**Corollary 2.** *In the decentralized supply chain with a fair-minded retailer*, *we have*

(i) $\partial E\pi_{m\lambda }^{D*}/\partial \lambda <0$, $\partial EU_{r\lambda }^{*}/\partial \lambda <0$ and $\partial E\pi_{sc\lambda }^{D*}/\partial \lambda <0$;

(ii)If *β*^2^/*k*<4*α*/3, then $\partial E\pi_{r\lambda }^{*}/\partial \lambda >0$; If *β*^2^/*k*>4*α*/3 and *λ*<(4*kα*−2*β*^2^)/(3*β*^2^−4*kα*), then $\partial E\pi_{r\lambda }^{*}/\partial \lambda >0$; If *β*^2^/*k*>4*α*/3 and *λ*>(4*kα*−2*β*^2^)/(3*β*^2^−4*kα*), then $\partial E\pi_{r\lambda }^{*}/\partial \lambda <0$.

Corollary 2 indicates that the expected profits of both the manufacturer and total supply chain are always negatively correlated with the retailer’s fairness concern coefficient. When *β*^2^/*k*≤4*α*/3, namely, the efficiency of energy-saving investment is low, the expected profit of the retailer is increasing in the fairness concern coefficient. In addition, the expected profit of the retailer is decreasing in the fairness concern coefficient when *β*^2^/*k*>4*α*/3 and *λ*>(4*kα*−2*β*^2^)/(3*β*^2^−4*kα*) hold synchronously, whereas the expected profit of the retailer is also increasing in the fairness concern coefficient when *β*^2^/*k*>4*α*/3 and *λ*<(4*kα*−2*β*^2^)/(3*β*^2^−4*kα*) hold synchronously. The above analysis implies that the fairness behavior of the retailer always causes the manufacturer and total supply chain to suffer, while the retailer can always benefit from the fairness behavior when the efficiency of energy-saving investment is low. It is worth noting that when the efficiency of energy-saving investment is high, with intensified fairness concern, the expected profit of the retailer goes up first and then goes down. In sum, so long as the retailer keeps moderate fairness concern level, the fairness behavior benefits it all the time. When the fairness behavior is intensified, the fairness behavior benefits the retailer when and only when the efficiency of energy-saving investment is low. Moreover, although the retailer can benefit from the intensified fairness, the associated utility of the retailer is always decreased.

Comparing with the fair-neutral scenario, the expected profits of both the manufacturer and total supply chain are lower under the fair-minded scenario, while the expected profit of the retailer may be higher or lower. Let *λ* = 0, we get the results of the fair-neutral scenario, the optimal expected profits of the manufacturer and retailer are characterized as $E\pi_{mn}^{D*}=k{\left(a-\alpha c\right)}^{2}/\left(8k\alpha -2\beta^{2}\right)$ and $E\pi_{rn}^{D*}=k^{2}\alpha {\left(a-\alpha c\right)}^{2}/{\left(4k\alpha -\beta^{2}\right)}^{2}$, respectively. Then the optimal expected profit of the total supply chain is characterized as $E\pi_{scn}^{D*}=k(6k\alpha -\beta^{2}){\left(a-\alpha c\right)}^{2}/[2{\left(4k\alpha -\beta^{2}\right)}^{2}]$.

Further, conducting the comparison analysis, we derive the following corollary.

**Corollary 3.** *Conducting the comparison analysis*, *we have*

(i) $e^{I*}>e_{n}^{D*}>e_{\lambda }^{D*}$, $E\pi_{sc}^{I*}>E\pi_{scn}^{D*}>E\pi_{sc\lambda }^{D*}$.

(ii) if 0<*β*^2^/*k*<2*α*(1+*λ*)/(2+3*λ*), then $p_{n}^{D*}>p_{\lambda }^{D*}>p_{}^{I*}$; if 2*α*(1+*λ*)/(2+3*λ*)<*β*^2^/*k*<*α*, then $p_{n}^{D*}>p_{}^{I*}>p_{\lambda }^{D*}$; if *β*^2^/*k*>*α*, then $p_{}^{I*}>p_{n}^{D*}>p_{\lambda }^{D*}$.

Corollary 3 shows that both the energy-saving level and the expected profit of the total supply chain in the decentralized system are always lower than that in centralized system. The retail price in the decentralized system may be lower or more than that in the centralized system, it depends on the relationships among the efficiency of energy-saving investment, the fairness concern parameter of the retailer and the consumer’s sensitivity parameter in retail price.

Let $A_{e\lambda }=e^{I*}-e_{\lambda }^{D*}$, we get ∂*A*_*eλ*_/∂*λ* = 4*kαβ*(*a*−*αc*)/[4*kα*(1+*λ*)−*β*^2^]^2^>0. Similarly, let $A_{\pi \lambda }=E\pi_{sc}^{I*}-E\pi_{sc\lambda }^{D*}$, we get ∂*A*_*πλ*_/∂*λ* = 4*k*^2^*αβ*^2^(*a*−*αc*)^2^(1+3*λ*)/[4*kα*(1+*λ*)−*β*^2^]^3^>0. Thus, as the retailer’s fairness behaviour is intensified, the gap continues to widen. This can be explained by two aspects: double marginalization and the effect of fairness behaviour. Therefore, establishing a cooperative relationship between the manufacturer and the retailer to mitigate the adverse impact of the retailer’s fairness behaviour and double marginalization will benefit the manufacturer and the total supply chain, as well as environmental sustainability. In addition, the retailer can also benefit from cooperative relationship by appropriate distribution of profits.

### Risk aversion scenario

In this subsection, we investigate the scenario of the supply chain with a retailer exhibiting risk-averse characteristic. The retailer is conservative about demand volatility, and aims to maximize its utility. Similar to the literature [20, 38, 42], the mean-variance function that is applied to characterize the risk aversion behavior and assess the retailer’s utility, is given as

$$U_{r\eta }^{D}=E\pi_{r}^{D}-\eta Var(\pi_{r}^{D})$$ (22)

where $Var(\pi_{r}^{D})$ is the variance of the retailer’s profit, *η*(*η*>0) is the risk aversion coefficient of the retailer and represents the risk attitude towards the demand uncertainty. The greater the risk aversion coefficient is, the more risk-averse the retailer is. *η* = 0 corresponds to the risk-neutral scenario. Therefore, in the mean-variance theory, the objective of the retailer is to maximize the expected profit minus the product of the risk aversion coefficient and the variance of profit. Moreover, the retailer can control its risk via the risk aversion coefficient to adjust the difference between the expected profit and the variance of profit.

Thus, when the retailer concerns demand volatility and exhibits risk-averse characteristic, the objective function of the retailer is

$$EU_{r\eta }^{D}=E\pi_{r}^{D}-\eta {\left(p_{\eta }^{D}-w_{\eta }^{D}\right)}^{2}\sigma^{2}$$ (23)

Substituting Eq (11) into the above equation, we get

$$EU_{r\eta }^{D}=(p_{\eta }^{D}-w_{\eta }^{D})[a+\beta e_{\eta }^{D}+\eta \sigma^{2}w_{\eta }^{D}+\lambda c-p_{\eta }^{D}(\alpha +\eta \sigma^{2})]$$ (24)

**Proposition 2.** *In the decentralized supply chain with a risk-averse retailer*, *the optimal energy-saving level and wholesale price of the manufacturer are*

$$e_{\eta }^{D*}=\frac{\left(a-\alpha c)\beta (\alpha +2\eta \sigma^{2}\right)}{4\alpha k(\alpha +\eta \sigma^{2})-\beta^{2}(\alpha +2\eta \sigma^{2})}$$ (25)

$$w_{\eta }^{D*}=\frac{2ak(\alpha +\eta \sigma^{2})-c[2\alpha k(\alpha +\eta \sigma^{2})-\beta^{2}(\alpha +2\eta \sigma^{2})]}{4\alpha k(\alpha +\eta \sigma^{2})-\beta^{2}(\alpha +2\eta \sigma^{2})}$$ (26)

and the optimal retail price is

$$p_{\eta }^{D*}=\frac{ak(3\alpha +2\eta \sigma^{2})+c(\alpha k-\beta^{2})(\alpha +2\eta \sigma^{2})}{4\alpha k(\alpha +\eta \sigma^{2})-\beta^{2}(\alpha +2\eta \sigma^{2})}$$ (27)

Proposition 2 shows that the expected profit of the manufacturer can be maximized at $e_{\eta }^{D*}$ and $w_{\eta }^{D*}$, and the retailer’s expected utility can be maximized at $p_{\eta }^{D*}$. Then, conducting the sensitivity and comparison analysis, we obtain the following corollaries.

**Corollary 4.** *In the decentralized supply chain with a risk-averse retailer*, *we have*

(i) $\partial e_{\eta }^{D*}/\partial \eta >0$, $\partial w_{\eta }^{D*}/\partial \eta >0$.

(ii) If 0<*β*^2^/*k*<*α*, then $\partial p_{\eta }^{D*}/\partial \eta <0$; if *α*<*β*^2^/*k*≤2*α*, then $\partial p_{\eta }^{D*}/\partial \eta >0$.

Corollary 4 shows that both the energy-saving level and the wholesale price are always increasing in the risk aversion degree of the retailer, and are more than that under the risk-neutral case. This implies that the risk aversion behavior could promote energy-saving investment, and further benefit the sustainability in the supply chain. It is noted that the retail price may be increasing or decreasing in the risk aversion degree of the retailer. In particular, the retail price is independent on the risk aversion degree when *β*^2^/*k* = *α* holds. Let *η* = 0, all the optimal energy-saving level, wholesale price and retail price are consistent with that under the fair-neutral scenario, the analysis verifies that the formulations in characterizing the fairness and risk behaviors of the retailer are effective, and our results are correct.

Furthermore, in the supply chain with a risk-averse retailer, the optimal expected profits of the manufacturer, retailer, and total supply chain can be given by

$$E\pi_{m\eta }^{D*}=\frac{k{\left(a-\alpha c\right)}^{2}(\alpha +2\eta \sigma^{2})}{8\alpha k(\alpha +\eta \sigma^{2})-2\beta^{2}(\alpha +2\eta \sigma^{2})}$$ (28)

$$E\pi_{r\eta }^{D*}=\frac{k^{2}\alpha^{2}{\left(a-\alpha c\right)}^{2}(\alpha +2\eta \sigma^{2})}{{\left[\beta^{2}(\alpha +2\eta \sigma^{2})-4\alpha k(\alpha +\eta \sigma^{2})\right]}^{2}}$$ (29)

$$E\pi_{sc\eta }^{D*}=E\pi_{m\eta }^{D*}+E\pi_{r\eta }^{D*}=\frac{k{\left(a-\alpha c\right)}^{2}(\alpha +2\eta \sigma^{2})[2\alpha k(3\alpha +2\eta \sigma^{2})-\beta^{2}(\alpha +2\eta \sigma^{2})]}{2{\left[\beta^{2}(\alpha +2\eta \sigma^{2})-4\alpha k(\alpha +2\eta \sigma^{2})\right]}^{2}}$$ (30)

The optimal expected utility of the risk-averse retailer is

$$EU_{r\eta }^{D*}=\frac{k^{2}\alpha^{2}{\left(a-\alpha c\right)}^{2}(\alpha +\eta \sigma^{2})}{{\left[\beta^{2}(\alpha +2\eta \sigma^{2})-4\alpha k(\alpha +\eta \sigma^{2})\right]}^{2}}$$ (31)

**Corollary 5.** *In the decentralized supply chain with a risk-averse retailer*, *we have*

(i) $\partial E\pi_{m\eta }^{*}/\partial \eta >0$, $\partial E\pi_{sc\eta }^{*}/\partial \eta >0$.

(ii) If 0<*β*^2^/*k*<4*αησ*^2^/(*α*+2*ησ*^2^), then $\partial E\pi_{r\eta }^{D*}/\partial \eta <0$ and $\partial EU_{r\eta }^{D*}/\partial \eta <0$; If 4*αησ*^2^/(*α*+2*ησ*^2^)<*β*^2^/*k*<4*α*(*α*+*ησ*^2^)/(3*α*+2*ησ*^2^), then $\partial E\pi_{r\eta }^{D*}/\partial \eta >0$ and $\partial EU_{r\eta }^{D*}/\partial \eta <0$; If 4*α*(*α*+*ησ*^2^)/(3*α*+2*ησ*^2^)<*β*^2^/*k*≤2*α*, then $\partial E\pi_{r\eta }^{D*}/\partial \eta >0$ and $\partial EU_{r\eta }^{D*}/\partial \eta >0$.

Corollary 5 shows the impact of the risk aversion behaviour of the retailer on the expected profits of both the parties and the total supply chain, as well as on the utility of the retailer. The expected profits of both the manufacturer and total supply chain are more than that under the risk-neutral case. The more risk-averse the retailer is, the more the expected profits of both the manufacturer and total supply chain are. That is, the risk aversion behaviour of the retailer could benefit the manufacturer and total supply chain. It’s worth noting that both the expected profit and utility of the retailer may be increasing or decreasing in the risk aversion degree, and this depends on the relationships among the efficiency of energy-saving investment, the risk aversion coefficient, the consumer’s sensitivity parameter in retail price and standard deviation of demand. Let *η* = 0, we get the results of the risk-neutral case, where all the optimal expected profits of the manufacturer, retailer and total supply chain are consistent with that under the fair-neutral scenario.

**Corollary 6.** *Compared with the centralized system*, *we have*

(i) $e^{I*}>e_{\eta }^{D*}>e_{n}^{D*}$, $E\pi_{sc}^{I*}>E\pi_{sc\eta }^{D*}>E\pi_{scn}^{D*}$.

(ii) If 0<*β*^2^/*k*<*α*, then $p_{n}^{D*}>p_{\eta }^{D*}>p_{}^{I*}$; if *α*<*β*^2^/*k*<2*α*, then $p_{}^{I*}>p_{\eta }^{D*}>p_{n}^{D*}$.

Corollary 6 shows that when the retailer exhibits risk-averse characteristic, both the energy-saving level and total expected profit of the supply chain in the decentralized system are always lower than that in the centralized system. Let $A_{e\eta }=e^{I*}-e_{\eta }^{D*}$ and $A_{\pi \eta }=E\pi_{sc}^{I*}-E\pi_{sc\eta }^{D*}$, we can get that the difference of energy-saving level between the decentralized system and the centralized system is decreasing in the risk aversion degree of the retailer since ∂*A*_*eη*_/∂*η* = −4*α*^2^*βσ*^2^*k*(*a*−*αc*)/[4*αk*(*α*+*ησ*^2^)−*β*^2^(*α*+2*ησ*^2^)]^2^<0. Also, the difference of expected profit between the decentralized system and the centralized system is decreasing in the risk aversion degree of the retailer since ∂*A*_*πη*_/∂*η* = −8*α*^4^*σ*^2^*k*^3^(*a*−*αc*)^2^/[4*αk*(*α*+*ησ*^2^)−*β*^2^(*α*+2*ησ*^2^)]^3^<0. In addition, the retail price in the decentralized system may be less or more than that in the centralized system. It depends on the relationships between the efficiency of energy-saving investment and the consumer’s sensitivity parameter in retail price. Corollary 6 reveals that the retailer can control its risk attitude to improve the profits of both the two channel members in the decentralized supply chain. Nevertheless, it is more beneficial to obtain the system-wide optimal expected profit of the supply chain by establishing a cooperative relationship between the manufacturer and the retailer, which is equal to that in the centralized system.

## Revenue-cost-sharing contract coordination model

In the previous section, it is verified that whatever behavioral concerns the retailer involves, both the environmental and commercial performances of the supply chain in the decentralized system are lower than that in the centralized system. In this section, we propose a revenue-cost-sharing contract to develop supply chain coordination models, which is widely used in conventional supply chains [47]. The revenue-cost-sharing contract is characterized by three parameters, namely wholesale price *w*, revenue-sharing coefficient *φ* and cost-sharing coefficient *θ*. Under which the retailer earns *φ* portion of the revenue of per unit product and the other (1−*φ*) portion of per unit revenue belongs to the manufacturer. Meanwhile, the retailer agrees to bear a fraction *θ* of energy-saving investment cost as well. Next, we still analyze the optimal strategy for each member with a Stackelberg game in which the manufacturer acts as the leader and the retailer is the follower.

Under the revenue-cost-sharing contract, the profit functions of the manufacturer and the retailer are given by

$$\pi_{m}^{R}=\left[(1-\varphi )p^{R}+w-c\right](\tilde{a}-\alpha p^{R}+\beta ep^{R})-\frac{1}{2}k(1-\theta ){\left(ep^{R}\right)}^{2}$$ (32)

$$\pi_{r}^{R}=(\varphi p^{R}-w)(\tilde{a}-\alpha p^{R}+\beta e^{R})-\frac{1}{2}k\theta {\left(e^{R}\right)}^{2}$$ (33)

When the retailer involves fairness concern, given any fixed $e_{{}_{\lambda }}^{R}$, the expected utility of the retailer is

$$EU_{r\lambda }^{R}=[(\varphi +\lambda \varphi -\lambda )p_{{}_{\lambda }}^{R}-(1+\lambda )w+\lambda c](a-\alpha p_{{}_{\lambda }}^{R}+\beta e_{{}_{\lambda }}^{R})-\frac{1}{2}\lambda k(\theta -\lambda +\lambda \theta ){\left(e_{{}_{\lambda }}^{R}\right)}^{2}$$ (34)

**Proposition 3.** *When the retailer involves fairness concern*, *under the revenue-cost-sharing contract*, *the optimal energy-saving level is*

$$e_{\lambda }^{R*}=\frac{\beta \left(a-c\alpha +w\alpha -a\varphi \right)}{2k\alpha -\beta^{2}-2k\alpha \theta +\beta^{2}\varphi }$$ (35)

and the optimal retail price is

$$\begin{array}{l} p_{\lambda }^{R*}=\frac{2k\left(\theta -1\right)\left[w\alpha \left(1+\lambda \right)+a\left(\lambda \varphi +\varphi -\lambda \right)-c\alpha \lambda \right]}{2\left[2k\alpha \left(\theta -1\right)-\beta^{2}\left(\varphi -1\right)\right]\left(\lambda \varphi +\varphi -\lambda \right)}\\ \;+\frac{\beta^{2}\left[w\left(1+2\lambda -2\lambda \varphi -2\varphi \right)+c\left(2\lambda \varphi +\varphi -2\lambda \right)\right]}{2\left[2k\alpha \left(\theta -1\right)-\beta^{2}\left(\varphi -1\right)\right]\left(\lambda \varphi +\varphi -\lambda \right)} \end{array}$$ (36)

Proposition 3 shows that both the optimal energy-saving level and retail price depend on the parameters of the revenue-cost-sharing contract. Thus, setting appropriate contract parameters as in Proposition 3 would induce the manufacturer to set the energy-saving level of the decentralized decision as that of the centralized one. Similarly, it can also induce the retailer to set the retail price of the decentralized decision as that of the centralized one. Let $e_{\lambda }^{R*}=e^{I*}$ and $p_{\lambda }^{R*}=p^{I*}$, we get *φ* = *θ* = *w*/*c*. That is, when *φ* = *θ* = *w*/*c*, the supply chain is coordinated and the system-wide optimal environmental and commercial performances are achieved under the revenue-cost-sharing contract. In this case, the optimal expected profits of the manufacturer and retailer can be characterized respectively as

$$E\pi_{m\lambda }^{R*}=\frac{k(c-w){\left(a-c\alpha \right)}^{2}}{c(4k\alpha -2\beta^{2})}=(1-\frac{w}{c})E\pi_{sc}^{I*}$$ (37)

$$E\pi_{r\lambda }^{R*}=\frac{kw{\left(a-c\alpha \right)}^{2}}{c(4k\alpha -2\beta^{2})}=\frac{w}{c}E\pi_{sc}^{I*}$$ (38)

This indicates that the system-wide optimal expected profit of the supply chain can be allocated arbitrarily between the manufacturer and the retailer by the revenue-cost-sharing contract. The manufacturer and the retailer just need to jointly confirm an appropriate wholesale price to obtain a win-win outcome. In this case, the revenue-cost-sharing contract is applicable in practice.

Further, when the retailer concerns demand volatility and exhibits risk-averse characteristic, given any fixed $e_{\lambda }^{R}$, the objective function of the retailer is

$$EU_{r\eta }^{R}=(\varphi p_{{}_{\eta }}^{R}-w)(a-\alpha p_{{}_{\eta }}^{R}+\beta e_{{}_{\eta }}^{R})-{\left(p_{{}_{\eta }}^{R}-w\right)}^{2}\eta \sigma^{2}-\frac{1}{2}\theta k{\left(e_{{}_{\eta }}^{R}\right)}^{2}$$ (39)

**Proposition 4.** *When the retailer exhibits risk-averse characteristic*, *under the revenue- cost-sharing contract*, *the optimal energy-saving level is*

$$e_{\eta }^{R*}=\frac{\beta w\left[\alpha^{2}\varphi^{2}+\alpha \eta \sigma^{2}(1+2\varphi )-2\eta^{2}\sigma^{4}(\varphi -2)\right]-\beta (2\eta \sigma^{2}+\alpha \varphi )\left[a\varphi (\varphi -1)+c(\eta \sigma^{2}+\alpha \varphi )\right]}{2k(1-\theta ){\left(\eta \sigma^{2}+\alpha \varphi \right)}^{2}-\beta^{2}\varphi (1-\varphi )(2\eta \sigma^{2}+\alpha \varphi )}$$ (40)

and the optimal retail price is

$$p_{\eta }^{R*}=\frac{2k(1-\theta )(\eta \sigma^{2}+\alpha \varphi )(w\alpha +2\eta \sigma^{2}w+a\varphi )-\beta^{2}\varphi (2\eta \sigma^{2}c+\alpha \varphi c+\alpha w-2\alpha \varphi w-2\eta \sigma^{2}\varphi w)}{4k(1-\theta ){\left(\eta \sigma^{2}+\alpha \varphi \right)}^{2}-2\beta^{2}(1-\varphi )\varphi (2\eta \sigma^{2}+\alpha \varphi )}$$ (41)

Proposition 4 characterizes the optimal equilibriums of supply chain members when the retailer involves risk aversion. Following similar analysis, let $e_{\eta }^{R*}=e^{I*}$ and $p_{\eta }^{R*}=p^{I*}$, which generates the coordination condition of the supply chain with a risk-averse retailer.

## Numerical analysis

In this section, we use several numerical experiments to verify the theoretical analysis obtained in the previous sections, and mainly illustrate how the behavioral concerns affect the energy-saving and pricing decisions as well as corresponding expected profits. The specific values for the basic parameters are as follows: *a* = 100, *c* = 10, *α* = 1, *σ* = 2. In order to better examine our theoretical results, we set two types of energy-saving investment efficiency: the low efficiency is *β*^2^/*k* = 0.8 (*β* = 2,*k* = 5), and the high efficiency is *β*^2^/*k* = 1.25 (*β* = 2.5,*k* = 5).

First of all, we examine the optimal energy-saving level, retail price and total expected profit of the supply chain without behavioral concerns. Table 2 shows that, no matter the efficiency of energy-saving investment is low or high, both the optimal energy-saving level and total expected profit of the supply chain in the decentralized system are always lower than that in centralized system. However, the optimal retail price in the decentralized system may be lower or more than that in the centralized system. When the efficiency of energy-saving investment is low, the optimal retail price in the decentralized system is more than that in the centralized system, while the optimal retail price in the decentralized system is less than that in the centralized system when the efficiency of energy-saving investment is high. It’s worth noting that, when the efficiency of energy-saving investment increases, both the optimal energy-saving level and total expected profit of the supply chain in the decentralized system are not increasing as fast as that in centralized system, which generates that the difference in energy-saving level and total expected profit between the decentralized system and the centralized system increases. This implies that it is even more necessary to develop a cooperative relationship between the manufacturer and the retailer so as to achieve higher environmental and economic performances when the efficiency of energy-saving investment increases.

**Table 2:** Optimal decisions and expected profits in the centralized system and decentralized system.

investment efficiency	*e*^I*^	*p*^I*^	$E\pi_{sc}^{I*}$	$e_{n}^{D*}$	$p_{n}^{D*}$	$E\pi_{scn}^{D*}$
low	30	85	3375	11.3	94.4	2056.6
high	60	130	5400	16.4	108.2	2543.8

**Fig 1:**
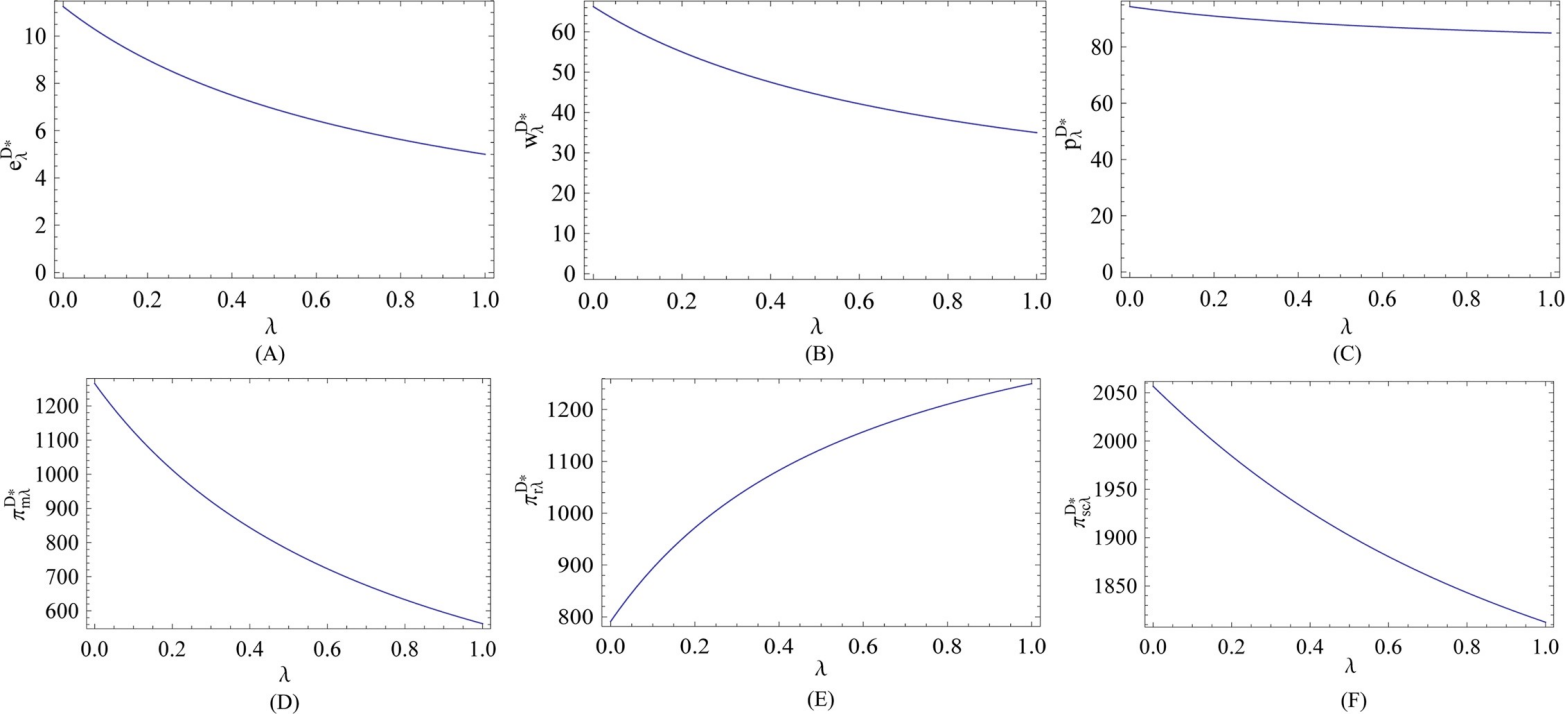
Effect of *λ* on the optimal decisions and corresponding expected profits (low efficiency).

**Fig 2:**
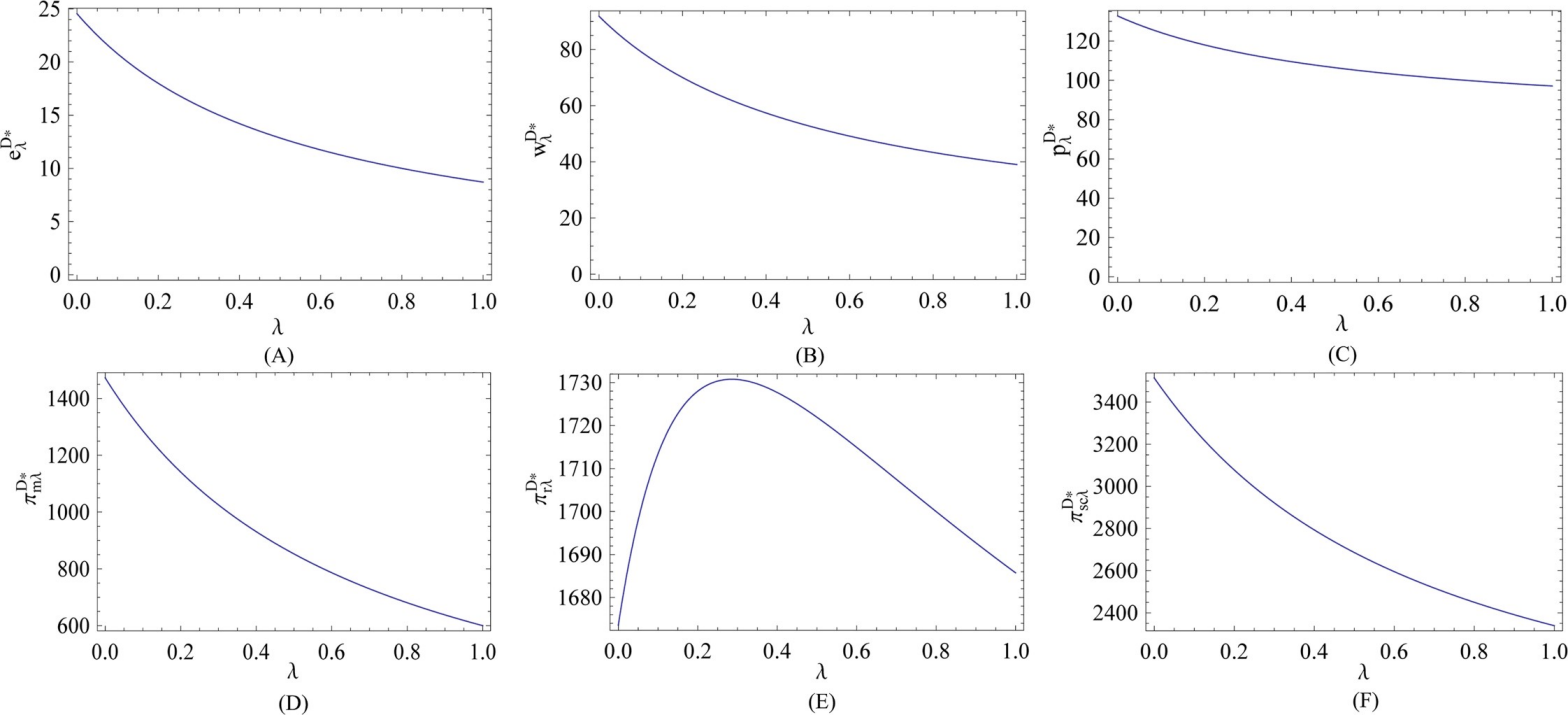
Effect of *λ* on the optimal decisions and corresponding expected profits (high efficiency).

In what follows, we visually illustrate the impact of the fairness behavior on the optimal decisions and corresponding expected profits in the decentralized system. As we can see from Figs 1 and 2, no matter the efficiency of energy-saving investment is low or high, all the optimal energy-saving level, wholesale price and retail price are decreasing in the fairness concern coefficient, and are lower than that in the fairness-neutral case. Also, we see that the retailer’s fairness behavior always reduces the manufacturer’s profit. Simultaneously, compared with the retailer, the manufacturer is more sensitive to the fairness concern coefficient in term of profits, and thereby the profit of the total supply chain is always negatively correlated with the retailer’s fairness concern coefficient. This implies that the retailer’s fairness behavior causes a negative effect on the manufacturer, total supply chain, and energy conservation. Therefore, when the retailer’s fairness concern is at a high level, developing a cooperative relationship between the manufacturer and the retailer is more beneficial for the manufacturer and supply chain system in terms of the energy-saving level per unit product and profits.

From Fig 1, we can see that when the efficiency of energy-saving investment is low, the retailer can always earn more in profits no matter what level the retailer’s fairness concern is at. However, we see from Fig 2 that when the efficiency of energy-saving investment is high, the retailer must keep moderate fairness concern level to earn more in profits. When the efficiency of energy-saving investment is high, as the fairness behavior is intensified, although the retailer’s share of the channel’s profit increases, the retailer also suffers a lot of loss under such circumstances. This implies that it is very necessary for the retailer to take the efficiency of energy-saving investment into consideration to keep reasonable level of fairness concern, because the retailer’s fairness behavior would also reduce its profit when the efficiency of energy-saving investment is high. All the results are consistent with the theoretical analyses in Subsection 3.1.

**Fig 3:**
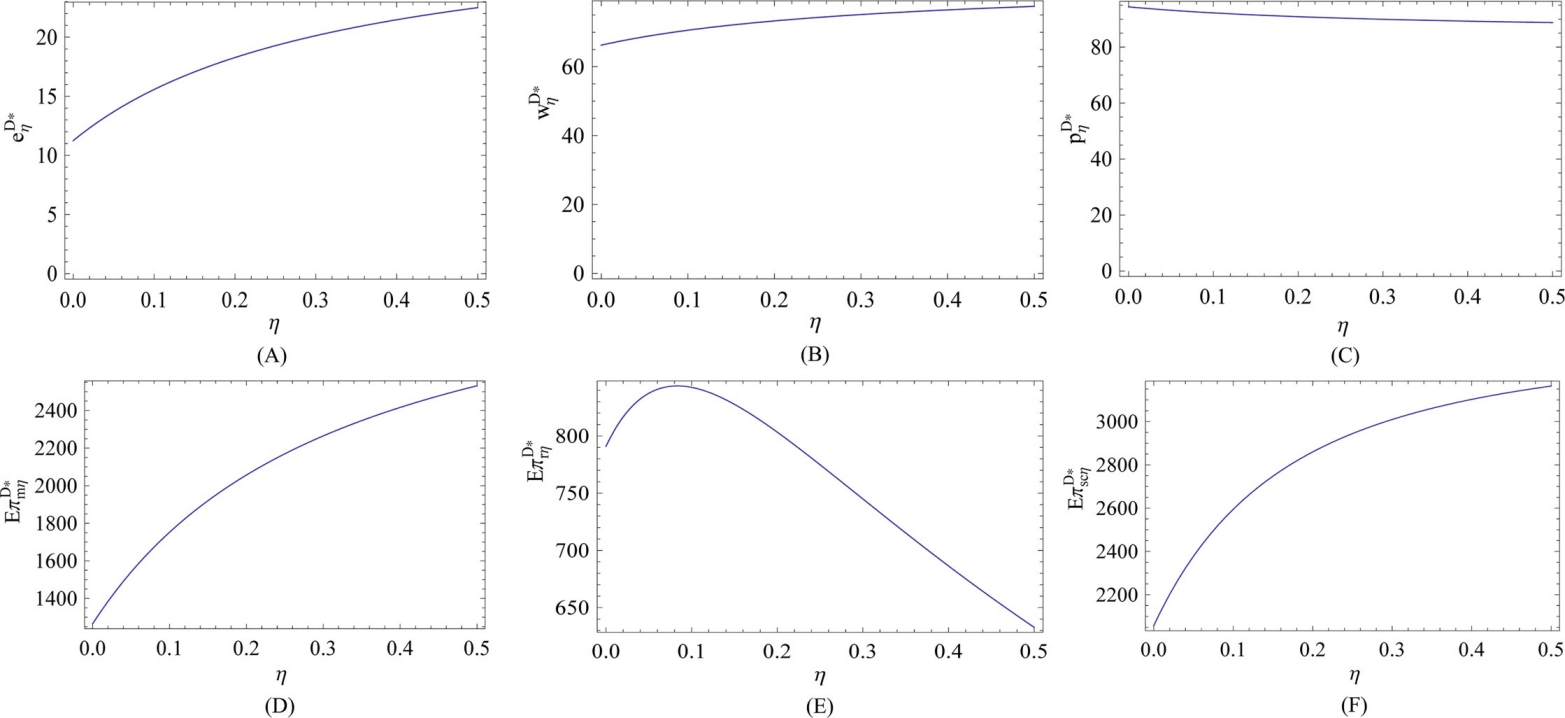
Effect of *η* on the optimal decisions and corresponding expected profits (low efficiency).

**Fig 4:**
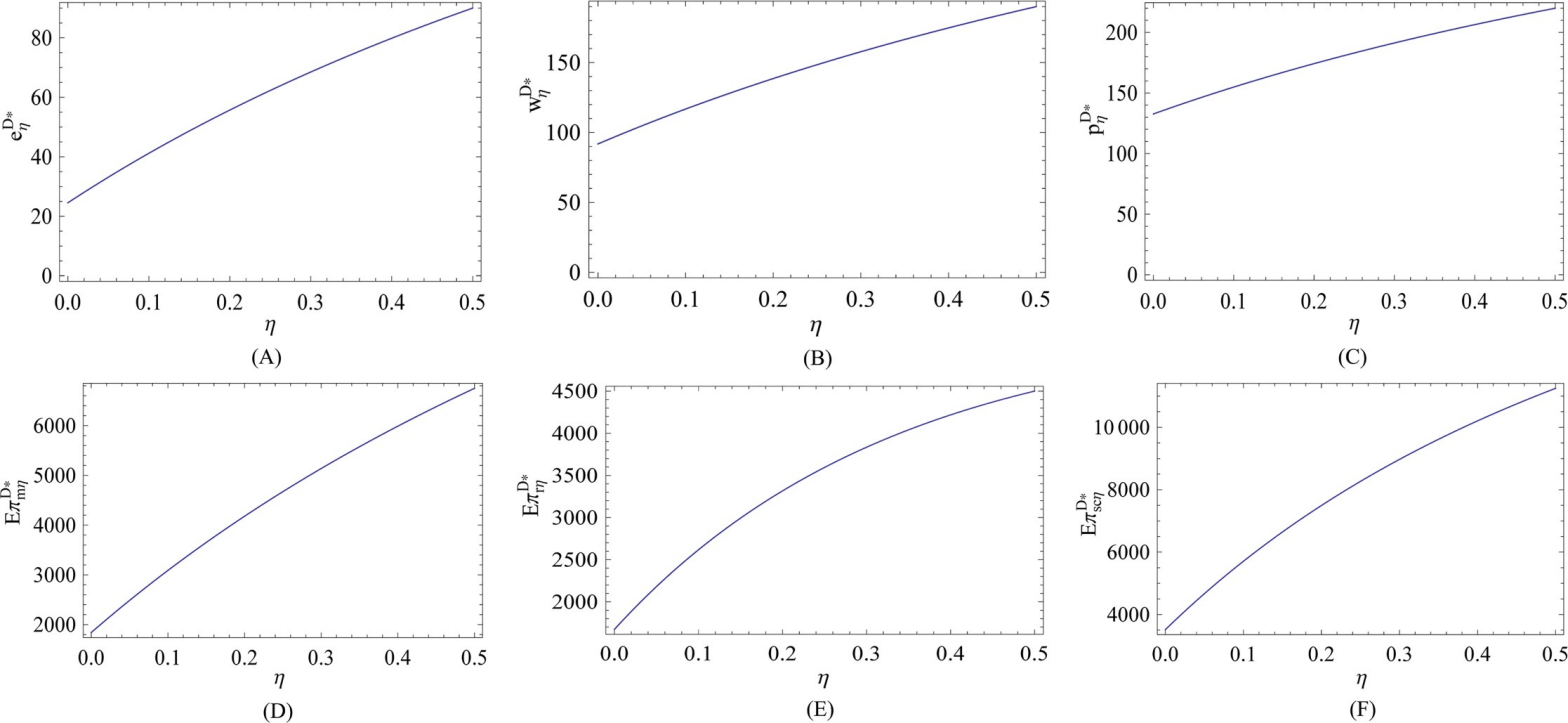
Effect of *η* on the optimal decisions and corresponding expected profits (high efficiency).

Lastly, we depict the impact of the risk aversion behavior on the optimal decisions and corresponding expected profits in the decentralized system. We see from Figs 3 and 4 that no matter the efficiency of energy-saving investment is low or high, both the energy-saving level and wholesale price are increasing in the risk aversion coefficient, and are higher than that in the risk-neutral case. It is interesting that when the efficiency of energy-saving investment is low, the retail price is decreasing in the risk aversion coefficient, whereas the retail price is increasing in the risk aversion coefficient when the efficiency of energy-saving investment is high. That is because when the efficiency of energy-saving investment is low, the retailer has to lower retail price to increase market demand and to reduce risk of overstock. When the efficiency of energy-saving investment is high, the manufacturer can invest more in energy-saving to attract more customers and to earn more profits. The energy-saving investment is costly, so the manufacturer will raise the wholesale price, and the retailer accordingly charges more per unit product from customer.

We also see that no matter the efficiency of energy-saving investment is low or high, the retailer’s risk aversion behavior can always benefit the manufacturer and total supply chain in term of profits, and the retailer can earn more profits when and only when the risk aversion is at a low level. It should be noted that although the risk aversion behavior can lead to greater energy-saving level and profits, as we have proved previously, it is still necessary to developing a cooperative relationship between the manufacturer and the retailer so as to improve energy-saving level and earn more profits.

## Conclusions

As is well-known, environmental pollution and energy shortages have become increasingly prominent. Meanwhile, there is no doubt that managers in today’s many industries may have different behavioral concerns when they make decisions, which significantly affect the economic and environmental performances of supply chains. In this paper, we consider a two-stage supply chain consisting of a socially responsible manufacturer and a retailer involving behavioral concerns in a single period, where the manufacturer produces energy-saving products and sells them to the retailer, and then the retailer resells energy-saving products to end consumers in the selling season. We first use the centralized system to establish a performance benchmark, and obtain the optimal energy-saving level, retail price and corresponding profit. Then, we explore the optimal strategies for the two channel members in the decentralized system, and formulate the problem as a Stackelberg game in which the manufacturer acts as the leader and the retailer is the follower. We deduce the optimal equilibriums under different scenarios via backward induction. Through conducting the sensitivity and comparison analysis, we discuss the effect of the behavioral concerns on the optimal equilibriums and corresponding profits in detail. In order to improve the economic and environmental performances of the supply chain, a revenue-cost-sharing contract is proposed to coordinate the supply chain, under which the manufacturer and the retailer share profits and cost of the energy-saving investment with each other, thereby a win-win outcome is achieved and the energy-saving level is improved.

Several managerial implications can be derived through our analytical and numerical results. First, supply chain managers should pay more attention to the behavioral concerns as far as possible, since they not only generally exist at individual level or organizational level but also significantly affect the economic and environmental performances of supply chains. The optimal energy-saving levels and corresponding profits under behavioral concerns differ from that in the perfectly rational scenario. Second, the efficiency of energy-saving investment plays a very critical role in energy-saving and pricing decisions. The change of the optimal equilibriums with respect to the coefficients of behavioral concerns depends on the efficiency of energy-saving investment. For example, when the efficiency of energy-saving investment is low, the retailer can always earn more in profits no matter what level the fairness concern is at. However, the retailer may suffer when the efficiency of energy-saving investment is high. Last but not the least, no matter what behavioral concerns the retailer involves, the optimal energy-saving level and corresponding profits in the decentralized system are lower than that in the centralized system, thus it is always necessary to develop a cooperative relationship between the manufacturer and the retailer so as to achieve higher environmental and economic performances.

Future research could expand this study from several aspects. First, shipping is a sustainable transportation mode in terms of both energy saving and pollution prevention, following the existing literature [47–50], the problem in ship development with energy saving may be a potentially meaningful research direction. Second, this paper respectively considers the fairness concern and risk aversion, we in the future may formulate supply chain models considering the fairness concern and risk aversion simultaneously. Third, this paper only considers a two-stage supply chain consisting of a manufacturer and a retailer in a single period, thereby it would be an important extension of our models to consider multiple manufacturers/retailers or more complex supply chains. Last, we know that the energy-saving investment is costly, thus it is meaningful to consider the case where the manufacturer is constrained by capital.

## Supporting information

S1 Table Notation used in the model formulation. (DOCX)

S1 Fig Numerical analysis code. (DOCX)

S2 Fig Numerical analysis code. (DOCX)

S3 Fig Numerical analysis code. (DOCX)

S4 Fig Numerical analysis code. (DOCX)

S1 Appendix (DOCX)
