# Supplementary material for: Energy-saving and pricing decisions in a sustainable supply chain considering behavioral concerns
Source: PLoS One. 2020 Aug 4;15(8):e0236354. doi: 10.1371/journal.pone.0236354 (PMC7402509; doi:10.1371/journal.pone.0236354)
Supplement: S1 Table — (DOCX) [file pone.0236354.s001.docx]

**S1 Table. Notation used in the model formulation.**

**Table 1. Notations.**

| **Notation** | **Implication** |
| --- | --- |
| $c$ | production cost per unit of product |
| $w$ | wholesale price per unit of product |
| $p$ | retail price per unit of product |
| $e$ | energy-saving level |
| $\tilde{a}$ | potential intrinsic demand |
| $a$ | mean of the potential intrinsic demand |
| $\alpha$ | consumer’s sensitivity parameter in retail price |
| $\beta$ | consumer’s sensitivity parameter in energy-saving level |
| $\sigma$ | standard deviation of the potential intrinsic demand |
| $k$ | coefficient of energy-saving investment |
| $\lambda$ | fairness concern parameter of the retailer |
| $\eta$ | risk aversion degree of the retailer |
|  | revenue-sharing coefficient |
| $\theta$ | cost-sharing coefficient |
| $\pi$ | profit function |
| $U$ | utility function |
