# Supplementary material for: Energy-saving and pricing decisions in a sustainable supply chain considering behavioral concerns
Source: PLoS One. 2020 Aug 4;15(8):e0236354. doi: 10.1371/journal.pone.0236354 (PMC7402509; doi:10.1371/journal.pone.0236354)
Supplement: S2 Fig — (DOCX) [file pone.0236354.s003.docx]

**S2 Fig. Numerical analysis code.**

**{a = 100, \[Alpha] = 1, \[Beta] = 3, k = 5, \[Sigma] = 2, c = 10}**

**Plot[e = -(((-a + c *\[Alpha]) \[Beta])/(**

**4 k* \[Alpha] - \[Beta]^2 + 4 k* \[Alpha] *\[Lambda])), {\[Lambda],**

**0, 1.5}, AxesOrigin -> {0, 0}, Frame -> True,**

**FrameLabel -> {"\[Lambda]",**

**"\!\(\*SubsuperscriptBox[\(e\), \(\[Lambda]\), \(\(D\)\(*\)\)]\)"}]**

**Plot[w = -((**

**2 a* k + 2 c* k* \[Alpha] - c *\[Beta]^2 +**

**4 c* k* \[Alpha] *\[Lambda])/(-4 k* \[Alpha] + \[Beta]^2 -**

**4 k* \[Alpha] *\[Lambda])), {\[Lambda], 0, 1.5},**

**AxesOrigin -> {0, 0}, Frame -> True,**

**FrameLabel -> {"\[Lambda]",**

**"\!\(\*SubsuperscriptBox[\(w\), \(\[Lambda]\), \(\(D\)\(*\)\)]\)"}]**

**Plot[p = (-3 a k (1 + \[Lambda]) +**

**c (\[Beta]^2 - k \[Alpha] (1 + \[Lambda])))/(\[Beta]^2 -**

**4 k \[Alpha] (1 + \[Lambda])), {\[Lambda], 0, 1.5},**

**AxesOrigin -> {0, 0}, Frame -> True,**

**FrameLabel -> {"\[Lambda]",**

**"\!\(\*SubsuperscriptBox[\(p\), \(\[Lambda]\), \(\(D\)\(*\)\)]\)"}]**

**Plot[\[Pi]m = (**

**k (a - c \[Alpha])^2)/(-2 \[Beta]^2 +**

**8 k \[Alpha] (1 + \[Lambda])), {\[Lambda], 0, 1.5}, Frame -> True,**

**FrameLabel -> {"\[Lambda]",**

**"\!\(\*SubsuperscriptBox[\(\[Pi]\), \(m\[Lambda]\), \**

**\(\(D\)\(*\)\)]\)"}]**

**Plot[\[Pi]r = (**

**k^2 \[Alpha] (a - c \[Alpha])^2 (1 + \[Lambda]) (1 +**

**3 \[Lambda]))/(\[Beta]^2 -**

**4 k \[Alpha] (1 + \[Lambda]))^2, {\[Lambda], 0, 1.5},**

**Frame -> True,**

**FrameLabel -> {"\[Lambda]",**

**"\!\(\*SubsuperscriptBox[\(\[Pi]\), \(r\[Lambda]\), \**

**\(\(D\)\(*\)\)]\)"}]**

**Plot[(k (a - c \[Alpha])^2 (-\[Beta]^2 +**

**6 k \[Alpha] (1 + \[Lambda])^2))/(**

**2 (\[Beta]^2 - 4 k \[Alpha] (1 + \[Lambda]))^2), {\[Lambda], 0, 1.5},**

**Frame -> True,**

**FrameLabel -> {"\[Lambda]",**

**"\!\(\*SubsuperscriptBox[\(\[Pi]\), \(sc\[Lambda]\), \**

**\(\(D\)\(*\)\)]\)"}]**
