# Supplementary material for: Energy-saving and pricing decisions in a sustainable supply chain considering behavioral concerns
Source: PLoS One. 2020 Aug 4;15(8):e0236354. doi: 10.1371/journal.pone.0236354 (PMC7402509; doi:10.1371/journal.pone.0236354)
Supplement: S4 Fig — (DOCX) [file pone.0236354.s005.docx]

**S4 Fig. Numerical analysis code.**

**{a = 100, \[Sigma] = 2, c = 10, \[Alpha] = 1, \[Beta] = 3, k = 5}**

**Plot[e = -(((-a + c \[Alpha]) \[Beta] (\[Alpha] +**

**2 \[Eta] \[Sigma]^2))/(**

**4 k \[Alpha]^2 - \[Alpha] \[Beta]^2 +**

**4 k \[Alpha] \[Eta] \[Sigma]^2 -**

**2 \[Beta]^2 \[Eta] \[Sigma]^2)), {\[Eta], 0, 0.5},**

**AxesOrigin -> {0, 0}, Frame -> True,**

**FrameLabel -> {"\[Eta]",**

**"\!\(\*SubsuperscriptBox[\(e\), \(\[Eta]\), \(\(D\)\(*\)\)]\)"}]**

**Plot[w = -((-2 a k \[Alpha] - 2 c k \[Alpha]^2 +**

**c \[Alpha] \[Beta]^2 - 2 a k \[Eta] \[Sigma]^2 -**

**2 c k \[Alpha] \[Eta] \[Sigma]^2 +**

**2 c \[Beta]^2 \[Eta] \[Sigma]^2)/(**

**4 k \[Alpha]^2 - \[Alpha] \[Beta]^2 +**

**4 k \[Alpha] \[Eta] \[Sigma]^2 -**

**2 \[Beta]^2 \[Eta] \[Sigma]^2)), {\[Eta], 0, 0.5},**

**AxesOrigin -> {0, 0}, Frame -> True,**

**FrameLabel -> {"\[Eta]",**

**"\!\(\*SubsuperscriptBox[\(w\), \(\[Eta]\), \(\(D\)\(*\)\)]\)"}]**

**Plot[p = (**

**c (k \[Alpha] - \[Beta]^2) (\[Alpha] + 2 \[Eta] \[Sigma]^2) +**

**a k (3 \[Alpha] + 2 \[Eta] \[Sigma]^2))/(**

**4 k \[Alpha] (\[Alpha] + \[Eta] \[Sigma]^2) - \[Beta]^2 (\[Alpha] +**

**2 \[Eta] \[Sigma]^2)), {\[Eta], 0, 0.5}, AxesOrigin -> {0, 0},**

**Frame -> True,**

**FrameLabel -> {"\[Eta]",**

**"\!\(\*SubsuperscriptBox[\(p\), \(\[Eta]\), \(\(D\)\(*\)\)]\)"}]**

**Plot[\[Pi]m = (**

**k (a - c \[Alpha])^2 (\[Alpha] + 2 \[Eta] \[Sigma]^2))/(**

**8 k \[Alpha] (\[Alpha] + \[Eta] \[Sigma]^2) -**

**2 \[Beta]^2 (\[Alpha] + 2 \[Eta] \[Sigma]^2)), {\[Eta], 0, 0.5},**

**Frame -> True,**

**FrameLabel -> {"\[Eta]",**

**"\!\(\*SubsuperscriptBox[\(E\[Pi]\), \(m\[Eta]\), \**

**\(\(D\)\(*\)\)]\)"}]**

**Plot[\[Pi]r = (**

**k^2 \[Alpha]^2 (a - c \[Alpha])^2 (\[Alpha] +**

**2 \[Eta] \[Sigma]^2))/(-4 k \[Alpha] (\[Alpha] + \[Eta] \**

**\[Sigma]^2) + \[Beta]^2 (\[Alpha] + 2 \[Eta] \[Sigma]^2))^2, {\[Eta],**

**0, 0.5}, Frame -> True,**

**FrameLabel -> {"\[Eta]",**

**"\!\(\*SubsuperscriptBox[\(E\[Pi]\), \(r\[Eta]\), \**

**\(\(D\)\(*\)\)]\)"}]**

**Plot[(k (a - c \[Alpha])^2 (\[Alpha] +**

**2 \[Eta] \[Sigma]^2) (-\[Beta]^2 (\[Alpha] +**

**2 \[Eta] \[Sigma]^2) +**

**2 k \[Alpha] (3 \[Alpha] + 2 \[Eta] \[Sigma]^2)))/(**

**2 (-4 k \[Alpha] (\[Alpha] + \[Eta] \[Sigma]^2) + \[Beta]^2 \**

**(\[Alpha] + 2 \[Eta] \[Sigma]^2))^2), {\[Eta], 0, 0.5}, Frame -> True,**

**FrameLabel -> {"\[Eta]",**

**"\!\(\*SubsuperscriptBox[\(E\[Pi]\), \(sc\[Eta]\), \(\(D\)\(*\)\)]\**

**\)"}]**
