# Supplementary material for: Energy-saving and pricing decisions in a sustainable supply chain considering behavioral concerns
Source: PLoS One. 2020 Aug 4;15(8):e0236354. doi: 10.1371/journal.pone.0236354 (PMC7402509; doi:10.1371/journal.pone.0236354)
Supplement: S1 Appendix — (DOCX) [file pone.0236354.s006.docx]

Appendix A

**Proof of Proposition 1**

Given any fixed $e_{\lambda}^{D}$and $w_{\lambda}^{D}$, taking the first-order and second-order derivative of with respect to $p_{\lambda}^{D}$, we have

This implies that is a concave function of the retail price$p_{\lambda}^{D}$, hence there exists an optimal solution that will maximize the expected utility of the retailer. By the first-order optimality condition, the retailer’s optimal retail price is

$$p_{\lambda}^{D*}=\frac{a+(1+\lambda)\alpha w_{\lambda}^{D}-\lambda\alpha c+\beta e_{\lambda}^{D}}{2\alpha}$$

Substituting $p_{\lambda}^{D*}=\frac{a+(1+\lambda)\alpha w_{\lambda}^{D}-\lambda\alpha c+\beta e_{\lambda}^{D}}{2\alpha}$ into the expected profit function of the manufacturer, we have

$$E\pi_{m\lambda}^{D}=\frac{1}{2}(w_{\lambda}^{D}-c)[a+\beta e_{\lambda}^{D}+\lambda\alpha c-\alpha w_{\lambda}^{D}(1+\lambda)]-\frac{1}{2}k(e_{\lambda}^{D})^{2}$$

Differentiating with respect to $w_{\lambda}^{D}$and $e_{\lambda}^{D}$gives

$$\frac{\partial E\pi_{m\lambda}^{D}}{\partial w_{\lambda}}=\frac{1}{2}[a+\beta e_{\lambda}^{D}+\alpha c(1+2\lambda)-2\alpha w_{\lambda}^{D}(1+\lambda)]$$

$$\frac{\partial E\pi_{m\lambda}^{D}}{\partial e_{\lambda}^{D}}=\frac{1}{2}\beta(w_{\lambda}^{D}-c)-ke_{\lambda}^{D}$$

The Hessian matrix of $E\pi_{m\lambda}^{D}$ with respect to $w_{\lambda}^{D}$ and $e_{\lambda}^{D}$ is

$$H(w_{\lambda}^{D},e_{\lambda}^{D})=\left|\begin{matrix} \alpha(1+\lambda)\text{ }\frac{1}{2}\beta\\ \frac{1}{2}\beta\text{ }-k \end{matrix}\right|=\alpha k(1+\lambda)-\frac{1}{4}\beta^{2}$$

Since $2\alpha k-\beta^{2}>0$, we have $H(w_{\lambda}^{D},e_{\lambda}^{D})=\alpha k(1+\lambda)-\beta^{2}/4>0$. This implies that $E\pi_{m\lambda}^{D}$ is jointly concave in $w_{\lambda}^{D}$and $e_{\lambda}^{D}$, and there exists optimal solutions that will maximize the expected utility of the manufacturer. By the first-order optimality condition, we get Equation (15) and Equation (16). Substituting Equation (15) and Equation (16) into$p_{\lambda}^{D*}=\frac{a+(1+\lambda)\alpha w_{\lambda}^{D}-\lambda\alpha c+\beta e_{\lambda}^{D}}{2\alpha}$, we get Equation (17).

**Proof of Corollary 1**

Taking the first-order derivative of with respect to$\lambda$, we have

$$\frac{\partial e_{\lambda}^{D*}}{\partial\lambda}=-\frac{4\alpha k(a-\alpha c)\beta}{[\beta^{2}-4\alpha k(1+\lambda)]^{2}}<0$$

Similarly, we have

$$\frac{\partial w_{\lambda}^{D*}}{\partial\lambda}=-\frac{8\alpha k^{2}(a-\alpha c)}{[\beta^{2}-4\alpha k(1+\lambda)]^{2}}<0$$

$\frac{\partial p_{\lambda}^{D*}}{\partial\lambda}=-\frac{3k(a-\alpha c)\beta^{2}}{[\beta^{2}-4\alpha k(1+\lambda)]^{2}}<0$.

**Proof of Corollary 2**

Taking the first-order derivative of with respect to $\lambda$, we have

$$\frac{\partial E\pi_{m\lambda}^{D*}}{\partial\lambda}=-\frac{2\alpha k^{2}(a-\alpha c)^{2}}{[\beta^{2}-4\alpha k(1+\lambda)]^{2}}<0$$

Similarly, we have

$$\frac{\partial EU_{r\lambda}^{*}}{\partial\lambda}=\frac{k(a-\alpha c)^{2}\beta^{2}(\beta^{2}+8\alpha k\lambda)}{2[4\alpha k(1+\lambda)-\beta^{2}]^{3}}<0$$

$$\frac{\partial E\pi_{sc\lambda}^{D*}}{\partial\lambda}=-\frac{2\alpha k^{2}(a-\alpha c)^{2}\beta^{2}(1+3\lambda)}{[4\alpha k(1+\lambda)-\beta^{2}]^{3}}<0$$

$$\frac{\partial E\pi_{r\lambda}^{D*}}{\partial\lambda}=\frac{2\alpha k^{2}(a-\alpha c)^{2}[(4k\alpha-2\beta^{2})+\lambda(4k\alpha-3\beta^{2})]}{[4k\alpha(1+\lambda)-\beta^{2}]^{3}}$$

When $(4k\alpha-2\beta^{2})+\lambda(4k\alpha-3\beta^{2})>0$, namely, $\frac{\beta^{2}}{k}<\frac{4}{3}\alpha$, or $\frac{\beta^{2}}{k}>\frac{4}{3}\alpha$and $\lambda<\frac{4k\alpha-2\beta^{2}}{3\beta^{2}-4k\alpha}$, we get $\partial E\pi_{r\lambda}^{D*}/\partial\lambda>0$. When $(4k\alpha-2\beta^{2})+\lambda(4k\alpha-3\beta^{2})<0$, namely, $\frac{\beta^{2}}{k}>\frac{4}{3}\alpha$and $\lambda>\frac{4k\alpha-2\beta^{2}}{3\beta^{2}-4k\alpha}$, we get $\partial E\pi_{r\lambda}^{D*}/\partial\lambda<0$.

**Proof of Corollary 3**

From Corollary 1, we have $e_{n}^{D*}>e_{\lambda}^{D*}$, $E\pi_{scn}^{D*}>E\pi_{sc\lambda}^{D*}$ and$p_{n}^{D*}>p_{\lambda}^{D*}$. Since

$$e^{I*}-e_{n}^{D*}=\frac{2\alpha\beta k(a-\alpha c)}{(2\alpha k-\beta^{2})(4\alpha k-\beta^{2})}>0$$

$$E\pi_{sc}^{I*}-E\pi_{scn}^{D*}=\frac{2\alpha^{3}k^{3}(a-\alpha c)^{2}}{(2\alpha k-\beta^{2})(4\alpha k-\beta^{2})^{2}}>0$$

$$p^{I*}-p_{n}^{D*}=-\frac{2k(a-\alpha c)(\alpha k-\beta^{2})}{(2\alpha k-\beta^{2})(4\alpha k-\beta^{2})}$$

we get $e^{I*}>e_{n}^{D*}>e_{\lambda}^{D*}$ and $E\pi_{sc}^{I*}>E\pi_{scn}^{D*}>E\pi_{sc\lambda}^{D*}$*.* When $k\alpha-\beta^{2}>0$, we get $p^{I*}<p_{n}^{D*}$, otherwise, we get $p^{I*}>p_{n}^{D*}$. Further,

$$p^{I*}-p_{\lambda}^{D*}=-\frac{k(a-\alpha c)[2\alpha k(1+\lambda)-\beta^{2}(2+3\lambda)]}{(2\alpha k-\beta^{2})[4\alpha k(1+\lambda)-\beta^{2}]}$$

When $2k\alpha(1+\lambda)-\beta^{2}(2+3\lambda)>0$, we get $p_{\lambda}^{D*}>p^{I*}$, otherwise, we get $p^{I*}>p_{\lambda}^{D*}$. In sum, if $0<\frac{\beta^{2}}{k}<\frac{2\alpha(1+\lambda)}{2+3\lambda}$, we get$p_{n}^{D*}>p_{\lambda}^{D*}>p^{I*}$; if $\frac{2\alpha(1+\lambda)}{2+3\lambda}<\frac{\beta^{2}}{k}<\alpha$, we get$p_{n}^{D*}>p^{I*}>p_{\lambda}^{D*}$; if $\frac{\beta^{2}}{k}>\alpha$, we get$p^{I*}>p_{n}^{D*}>p_{\lambda}^{D*}$.

**Proof of Proposition 2**

Given any fixed $e_{\eta}^{D}$and $w_{\eta}^{D}$, Taking the first-order and second-order derivative of with respect to $p_{\eta}^{D}$, we have

This implies that is a concave function of $p_{\eta}^{D}$. By the first-order optimality condition, the retailer’s optimal retail price is

$$p_{\eta}^{D*}=\frac{a+w_{\eta}^{D}(\alpha+2\eta\sigma^{2})+\beta e_{\eta}^{D}}{2(\alpha+\eta\sigma^{2})}$$

Substituting $p_{\eta}^{D*}=\frac{a+w_{\eta}^{D}(\alpha+2\eta\sigma^{2})+\beta e_{\eta}^{D}}{2(\alpha+\eta\sigma^{2})}$ into the expected profit function of the manufacturer, we have

$$E\pi_{m\eta}^{D}=\frac{1}{2(\alpha+\eta\sigma^{2})}(w_{\eta}^{D}-c)(a+\beta e_{\eta}^{D}+\alpha w_{\eta}^{D})(\alpha+2\eta\sigma^{2})-\frac{1}{2}k(e_{\eta}^{D})^{2}$$

Differentiating with respect to $w_{\eta}^{D}$and $e_{\eta}^{D}$gives

$$\frac{\partial E\pi_{m\eta}^{D}}{\partial w_{\eta}^{D}}=\frac{1}{2(\alpha+\eta\sigma^{2})}(a+\beta e_{\eta}^{D}+\alpha c-2\alpha w_{\eta}^{D})(\alpha+2\eta\sigma^{2})$$

$$\frac{\partial E\pi_{m\eta}^{D}}{\partial e_{\eta}^{D}}=\frac{1}{2(\alpha+\eta\sigma^{2})}\beta(w_{\eta}^{D}-c)(\alpha+2\eta\sigma^{2})-ke_{\eta}^{D}$$

The Hessian matrix of $E\pi_{m\eta}^{D}$ with respect to $w_{\eta}^{D}$ and $e_{\eta}^{D}$ is

$H(w_{\eta}^{D},e_{\eta}^{D})=\left|\begin{matrix} -\frac{\alpha(\alpha+2\eta\sigma^{2})}{\alpha+\eta\sigma^{2}}\text{ }\frac{\beta(\alpha+2\eta\sigma^{2})}{2(\alpha+\eta\sigma^{2})} \\ \frac{\beta(\alpha+2\eta\sigma^{2})}{2(\alpha+\eta\sigma^{2})}\text{ }-k \end{matrix}\right|=\frac{4\alpha k(\alpha+2\eta\sigma^{2})(\alpha+\eta\sigma^{2})-\beta^{2}(\alpha+2\eta\sigma^{2})^{2}}{4(\alpha+\eta\sigma^{2})^{2}}>0$This implies that $E\pi_{m\eta}^{D}$ is jointly concave in $w_{\eta}^{D}$ and $e_{\eta}^{D}$. By the first-order optimality condition, we get Equation (25) and Equation (26). Substituting Equation (25) and Equation (26) into$p_{\eta}^{D*}=\frac{a+w_{\eta}^{D}(\alpha+2\eta\sigma^{2})+\beta e_{\eta}^{D}}{2(\alpha+\eta\sigma^{2})}$, we get Equation (27).

**Proof of Corollary 4**

Taking the first-order derivative of with respect to $\eta$, we have

$$\frac{\partial e_{\eta}^{D*}}{\partial\eta}=\frac{4k\alpha^{2}\beta\sigma^{2}(a-\alpha c)}{[\beta^{2}(\alpha+2\eta\sigma^{2})-4\alpha k(\alpha+\eta\sigma^{2})]^{2}}>0$$

Similarly, we have

$$\frac{\partial w_{\eta}^{D*}}{\partial\eta}=\frac{2\alpha k\beta^{2}\sigma^{2}(a-\alpha c)}{[\beta^{2}(\alpha+2\eta\sigma^{2})-4\alpha k(\alpha+\eta\sigma^{2})]^{2}}>0$$

$$\frac{\partial p_{\eta}^{D*}}{\partial\eta}=-\frac{4\alpha k(a-\alpha c)(\alpha k-\beta^{2})\sigma^{2}}{[\beta^{2}(\alpha+2\eta\sigma^{2})-4\alpha k(\alpha+\eta\sigma^{2})]^{2}}$$

When $\alpha k-\beta^{2}>0$, namely,$\beta^{2}/k<\alpha$, we get $\partial p_{\eta}^{D*}/\partial\eta<0$. When$\alpha k-\beta^{2}<0$, namely,$\beta^{2}/k>\alpha$, we get $\partial p_{\eta}^{D*}/\partial\eta>0$.

**Proof of Corollary 5**

Taking the first-order derivative of with respect to $\eta$, we have

$$\frac{\partial E\pi_{m\eta}^{D*}}{\partial\eta}=\frac{2\alpha^{2}k^{2}\sigma^{2}(a-\alpha c)^{2}}{[\beta^{2}(\alpha+2\eta\sigma^{2})-4\alpha k(\alpha+2\eta\sigma^{2})]^{2}}>0$$

Similarly, we have

$$\frac{\partial E\pi_{sc\eta}^{D*}}{\partial\eta}=\frac{8\alpha^{4}k^{3}\sigma^{2}(a-\alpha c)^{2}}{[4\alpha k(\alpha+\eta\sigma^{2})-\beta^{2}(\alpha+2\eta\sigma^{2})]^{3}}>0$$

$$\frac{\partial E\pi_{r\eta}^{D*}}{\partial\eta}=-\frac{2\alpha^{2}k^{2}\sigma^{2}(a-\alpha c)^{2}[4\alpha k\eta\sigma^{2}-\beta^{2}(\alpha+2\eta\sigma^{2})]}{[4\alpha k(\alpha+\eta\sigma^{2})-\beta^{2}(\alpha+2\eta\sigma^{2})]^{3}}$$

$$\frac{\partial EU_{r\eta}^{D*}}{\partial\eta}=-\frac{\alpha^{2}k^{2}\sigma^{2}(a-\alpha c)^{2}[4\alpha k(\alpha+\eta\sigma^{2})-\beta^{2}(3\alpha+2\eta\sigma^{2})]}{[4\alpha k(\alpha+\eta\sigma^{2})-\beta^{2}(\alpha+2\eta\sigma^{2})]^{3}}$$

When $4\alpha k\eta\sigma^{2}-\beta^{2}(\alpha+2\eta\sigma^{2})>0$, we get $\partial E\pi_{r\eta}^{*}/\partial\eta<0$; when $4\alpha k\eta\sigma^{2}-\beta^{2}(\alpha+2\eta\sigma^{2})<0$, we get $\partial E\pi_{r\eta}^{*}/\partial\eta>0$. Further, when$4\alpha k(\alpha+\eta\sigma^{2})-\beta^{2}(3\alpha+2\eta\sigma^{2})>0$, we get $\partial EU_{r\eta}^{D*}/\partial\eta<0$, and when $4\alpha k(\alpha+\eta\sigma^{2})-\beta^{2}(3\alpha+2\eta\sigma^{2})<0$, we get$\partial EU_{r\eta}^{D*}/\partial\eta>0$. In sum, we can obtain Corollary 5.

**Proof of Corollary 6**

From Corollary 4 and Corollary 5, we have$e_{\eta}^{*}>e_{n}^{*}$ and $E\pi_{sc\eta}^{*}>E\pi_{scn}^{*}$. Meanwhile, when$0<\beta^{2}/k<\alpha$, we have$p_{n}^{*}>p_{\eta}^{*}$; when$\alpha<\beta^{2}/k\leq2\alpha$, we have $p_{n}^{*}<p_{\eta}^{*}$. Since

$$e^{I*}-e_{\eta}^{D*}=\frac{2k\alpha^{2}\beta(a-\alpha c)}{(2k\alpha-\beta^{2})[4k\alpha(\alpha+\eta\sigma^{2})-\beta^{2}(\alpha+2\eta\sigma^{2})]}>0$$

$$E\pi_{sc}^{I*}-E\pi_{sc\eta}^{D*}=\frac{2k^{3}\alpha^{4}(a-\alpha c)^{2}}{(2k\alpha-\beta^{2})[4k\alpha(\alpha+\eta\sigma^{2})-\beta^{2}(\alpha+2\eta\sigma^{2})]^{2}}>0$$

$$p^{I*}-p_{\eta}^{D*}=-\frac{2k(a-\alpha c)(k\alpha-\beta^{2})}{(2k\alpha-\beta^{2})[4k\alpha(\alpha+\eta\sigma^{2})-\beta^{2}(\alpha+2\eta\sigma^{2})]}$$

We get $e^{I*}>e_{n}^{*}>e_{\lambda}^{*}$ and $\pi_{sc}^{I*}>\pi_{scn}^{*}>\pi_{sc\lambda}^{*}$. When $k\alpha-\beta^{2}>0$, we get $p^{I*}<p_{\eta}^{D*}$, otherwise, we get $p^{I*}>p_{\eta}^{D*}$. Thus, when$0<\beta^{2}/k<\alpha$, we get $p_{n}^{D*}>p_{\eta}^{D*}>p^{I*}$. And when $\alpha<\beta^{2}/k<2\alpha$, we get $p^{I*}>p_{\eta}^{D*}>p_{n}^{D*}$.

**Proof of Proposition 3**

Taking the first-order and second-order derivative of with respect to$p_{_{\lambda}}^{R}$, we have

When holds, then . This implies that is a concave function of the retail price$p_{_{\lambda}}^{R}$, hence there exists an optimal solution that will maximize the expected utility of the retailer. By the first-order optimality condition, the retailer’s optimal retail price is

Substituting into the expected profit function of the manufacturer, we have

Differentiating with respect to $e_{_{\lambda}}^{R}$gives

When holds, then . This implies that $E\pi_{m\lambda}^{R}$ is concave in $e_{\lambda}^{R}$, and there exists optimal solutions that will maximize the expected utility of the manufacturer. By the first-order optimality condition, we get Equation (34). Substituting Equation (34) into, we get Equation (35).

**Proof of Proposition 4**

Taking the first-order and second-order derivative of with respect to$p_{\eta}^{R}$, we have

This implies that is a concave function of the retail price$p_{_{\eta}}^{R}$, hence there exists an optimal solution that will maximize the expected utility of the retailer. By the first-order optimality condition, the retailer’s optimal retail price is

Substituting into the expected profit function of the manufacturer, we have

Differentiating with respect to $e_{\eta}^{R}$gives

When holds, then . This implies that $E\pi_{m\eta}^{R}$ is concave in $e_{\eta}^{R}$, and there exists optimal solutions that will maximize the expected utility of the manufacturer. By the first-order optimality condition, we get Equation (39) Substituting Equation (39) into, we get Equation (40).
